# Enhancing Outcomes for Outpatient Percutaneous Coronary Interventions

**DOI:** 10.1097/NUR.0000000000000334

**Published:** 2017-10-05

**Authors:** Kevin Spruce, Chondra Butler

**Affiliations:** **Author Affiliations:** Clinical Nurse Specialist (Dr Spruce), Surgical Services, Clovis Community Medical Center, CA; and Assistant Professor (Dr Butler), College of Nursing, University of South Alabama, Mobile.

**Keywords:** cardiac, nurse, outcomes, PCI, recovery

## Abstract

**Purpose and Objectives:**

A quality improvement project was conducted to create a sustainable continuum of care for increased volumes of outpatients receiving percutaneous coronary interventions. Dramatic growth exposed system vulnerabilities and left staff overwhelmed. Four postinterventional project objectives included reducing preprocedural preparation times, reducing bleeding complications, reducing hospital length of stay, and collectively increasing patient satisfaction.

**Description of the Project:**

Amidst creating a specialized postintervention coronary recovery area and acquiring and training existing preregistration and recovery nurses, a fragmented system of care was united. The clinical nurse specialist–led project used a systematic and evidence-based implementation process to harmoniously acclimate perioperative staff. An evaluation process further defined new opportunities to support a growing service line.

**Outcomes:**

Postimplementation data were collected over a 3-month period. An overall improvement was found in all targeted objectives, despite an upsurge in case volumes. A moderately significant correlation (*r* [105] = 0.424, *P* < .001) was found between bleeding occurrences and hospital length of stay.

**Conclusion:**

The synergy between interdepartmental collaboration and strategic staffing reallocation was shown to be invaluable to alleviate procedural areas of service, such as the cardiac catheterization laboratory. As a project champion, the clinical nurse specialist is an essential catalyst to identify and creatively surmount system-level challenges.

The provision of percutaneous coronary interventions (PCI) for patients with occlusive cardiac vascular disease has been a growing trend since the procedure debuted in the late 1970s.^1^ Subsequent advances in research, technology, and technique have placed PCI in the forefront of cardiac procedures, which have resulted in reduced levels of interventional risks and have supported expedient recoveries. However, the nursing continuum of care that ushers the patient through the PCI process can have a large impact on short- and long-term clinical outcomes.^2^

Preprocedural and postprocedural areas of service are instrumental gateways to safely receive and discharge patients, especially in the ambulatory setting. Preprocedural clinicians often face challenges to gather essential patient information to allow the interdisciplinary team to formulate a safe and effective perioperative plan of care.^3^ Likewise, postprocedural nurses frequently struggle to further coordinate patient care through a reciprocal loop of ineffective communication with the procedural team.^4^ A poor level of communication, compounded with a lack of nursing specialized recovery and discharge teaching skills, leave PCI patients discontented, vulnerable to detrimental complications, and at increased risk for poor health outcomes.^5^

Clinical nurse specialists (CNSs) who practice in the procedural setting can catalyze effective interdepartmental cohesion, especially when clinical resources are limited. Optimization of care across the PCI continuum should be accomplished in a manner that best suits the patient and supporting institution alike. As expert clinicians and agents of change, CNSs can lead quality improvement (QI) activities to produce cost-effective and long-term operational solutions.^6^

## BACKGROUND

The advent of new legal regulations often presents immense opportunities for clinical institutions to begin providing services to patients that were previously limited. California, for example, passed Senate Bill 906, allowing California hospitals without an on-site cardiac surgery program to provide elective PCI, providing they met specific criteria.^7^ Beginning in 2015, once hospitals and procedural centers became approved, patients had more potential locations for diagnostic and interventional services.

### System Problem

Senate Bill 906 created an opportunity for the patient population and a hospital located in central California. Consequently, cardiac catheterization laboratory personnel accustomed to supporting a relatively small number of emergent PCI began to feel the strain of an additional patient load. In the first year alone, diagnostic and interventional volumes doubled as the result of the new elective PCI program.^8^ The increased case load exposed a fragmented outpatient system of care in which existing nursing resources could not sustain optimal perioperative care.

From a systems perspective, 3 phases of care were negatively affected by the increased PCI volumes. The first phase of care was the patient telephone preregistration process. Previously, patient information such as health history, current medications, and allergies were entered into the electronic health record (EHR) before the patient arrived at the hospital. During phone preregistration, patients were also provided information to help prepare for their hospital arrival, such as to abstain from eating and drinking as appropriate. The cardiac catheterization laboratory staff typically performed this function. Because both inpatient and outpatient PCI volumes increased, the nurses in the laboratory could no longer devote enough time to thoroughly complete the outpatient preregistration process, thus leaving large EHR data gaps and more unprepared patients.

Over the course of 6 months, a second phase of care began to suffer setbacks from the diminishing preregistration resources. After telephone preregistration and subsequent on-site registration, patients were routed to an outpatient preprocedural preparation area called “Short Stay.” In this area, the medical chart was reviewed and the patient was prepared for their PCI procedure. As a result of growing EHR data gaps and last-minute efforts to obtain information, preprocedural preparation times increased by an average of 33%.^8^ Nurses working in Short Stay and their patients were becoming frustrated by the preparation delays. This situation resulted in a rushed process, thus increasing the risk for procedural and recovery complications due to the compromised quality of patient data.^3^

Postprocedural recovery represented the third phase of care that was affected by the increased outpatient PCI volumes. Under the previous recovery process, post-PCI patients were outsourced to the general postanesthesia care unit. Although the unit nurses were knowledgeable in post-PCI recovery, the increased number of patients placed a strain on nurses and revealed knowledge gaps related to managing PCI patients. Communication gaps also existed between postanesthesia unit and catheterization laboratory nurses, which led to delays in providing care and increased the risk of bleeding complications. The occurrence of procedural arterial access site bleeding complications also rose by 50% in a 6-month period.^8^

The culmination of the 3 severely strained phases of care ultimately resulted in negative patient feedback from procedural delays, an increased occurrence of complications, and increased lengths of hospital stays. Outpatient PCI satisfaction scores dropped, and an average of $18 000 in nonrecoverable complication-related fees were added each month.^9^–^11^ The formation of dedicated preregistration and recovery resources would become a value-added solution to boost patient safety, confidence, and satisfaction.

### Review of Literature

Through the patient-intervention-comparison-outcome process, a set of 4 elements was identified to guide a relevant electronic search of evidence-based literature.^12^ The patients were identified as individuals undergoing a PCI. The interventions represented the reallocation and subsequent training of existing nursing staff. A preintervention and postintervention comparison addressed the closure of resource gaps. Finally, patient satisfaction and bleeding outcomes would drive the success of the project. As a result, the following clinical question was formulated for a literature search: For PCI patients, does staffing reallocation and training close clinical gaps to reduce bleeding complications and increase patient satisfaction?

The final search results revealed and confirmed that there is a paucity of literature for the PCI continuum of care.^13^ As a result, the search parameters were widened to 8 years or newer to capture at least a level “2c” of evidence or higher, using the following search terms: “PCI,” “nurse,” “staffing,” and “outcomes.”^14^ Despite the limited results, there was a suitable amount of historical evidence to guide the project objectives.

The literature revealed that most cardiac catheterization laboratory environments experience process and data gaps.^4^ Communication, training, and staffing deficits precipitated the highest prevalence of vascular complications in PCI patients.^4^,^15^ The shifting of interdepartmental nursing roles, especially in preprocedural phases of care, can be effective to prevent detrimental data losses, improve collaboration, increase patient satisfaction, and improve long-term patient outcomes.^3^,^4^ Likewise, postanesthesia recovery nurses can be excellent resources to transition to specialized recovery areas.^3^,^15^

In the PCI recovery setting, close proximity to respective procedural areas can increase interprofessional collaboration and reduce postoperative complications.^4^,^5^ Congruently, reallocated staff should be indoctrinated into a training program and setting that focuses on learning consistent hemostasis and patient comfort techniques to increase patient satisfaction.^16^ Training in the recovery areas should also be validated through return demonstration to ensure that nurses can correlate acute clinical changes with impending complications.^17^–^19^ Finally, postdischarge teaching can be effectively conducted in the recovery setting. Percutaneous coronary intervention recovery nurses should become comfortable with one-on-one teaching sessions with their patients, where customized concerns can be addressed to promote a stronger sense of support and self-efficacy.^20^ An emphasis on postdischarge follow-up and education to prevent complications at home should also be provided to patients to mitigate short-term bleeding outcomes and long-term vascular complications.^21^

### Opportunities for Improvement

The author, a CNS at the hospital, saw an opportunity to use nursing resources within the whole perioperative continuum of care to solve the staffing shortage. As a result, a strengths-weaknesses-opportunities-threats analysis was conducted to visualize and cross-refer interdepartmental barriers with potential staffing reallocation benefits. The Table illustrates the following themes: cardiac catheterization laboratory staff needed support, preprocedural patient data gaps existed, initial interdepartmental discussions to support the laboratory staff–expressed hesitancy, there was an opportunity to reallocate recovery unit resources, there was a potential for recovery nurses' dissatisfaction after cross-training, successful sharing of preoperative and recovery tasks can develop interdepartmental ownership and cohesion, reallocated nurses need less training than extramural staff, and the institution could financially support the project.^22^ Evidence shows that reallocating interdepartmental staff is cost effective to improve PCI outcomes and is an excellent way to garner a higher level of stakeholder buy-in.^15^

### Project Purpose

The overarching purpose of this project was to transform an existing PCI program into a successful and sustainable endeavor with increased patient volume. Limited staffing in the presence of increasing patient growth demonstrated a decline in patient outcomes. There were 4 QI objectives that the CNS identified and vetted: (1) streamline and reduce preprocedural preparation times to close potentially harmful data gaps, (2) reduce costly PCI-related bleeding complications, (3) decrease the average amount of post-PCI recovery time and overall hospital length of stay for outpatients, and (4) improve patient satisfaction.

## QI MODEL

Quality improvement conceptually embodies an endeavor to refine a service, practice, or process to reach a higher state of efficiency, safety, and satisfaction.^23^ For this project, the services that provided care to PCI patients were not adequately staffed, and safety and satisfaction were being compromised. If an improvement was not made soon, the institution could not continue to offer long-term PCI services to the community.

The QI model that guided this project was the define-measure-analyze-improve-control (DMAIC) methodology. As part of Six Sigma tools, the DMAIC method was commonly used throughout the CNS's hospital system.^24^ Using a familiar approach with the project stakeholders was advantageous for obtaining buy-in.

**FIGURE 1.**
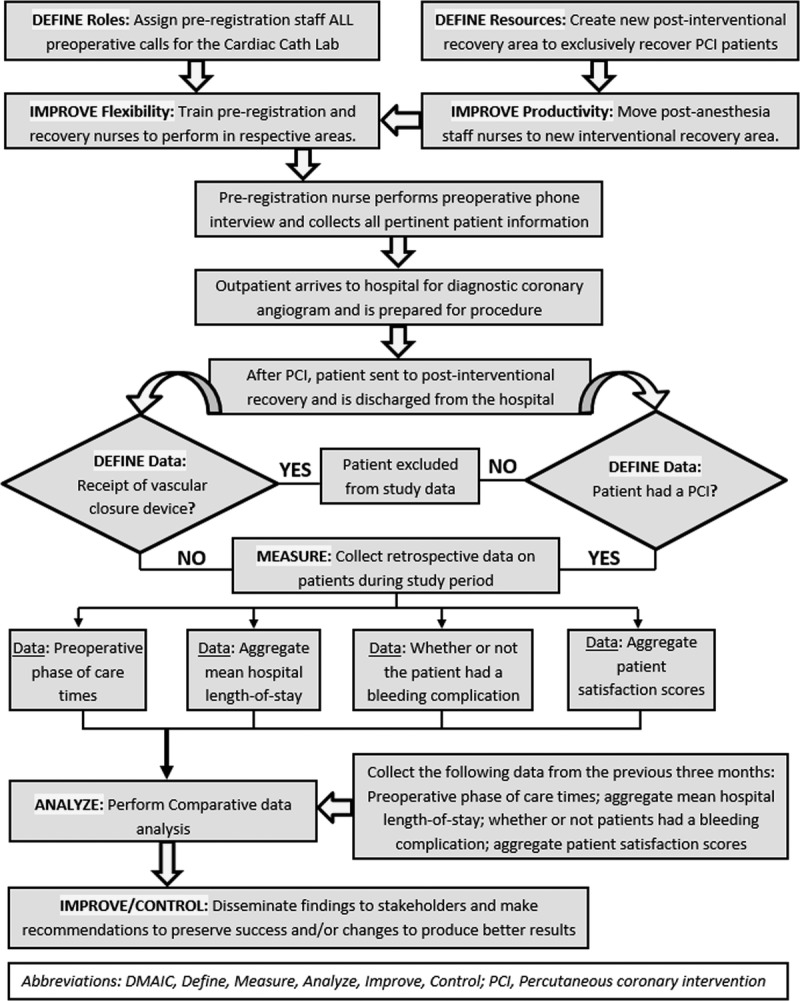
Project process map using DMAIC method. DMAIC, define, measure, analyze, improve, control; PCI, percutaneous coronary intervention.

The DMAIC method provided structure for the CNS to create a project process map (Figure 1). Initial steps allowed the CNS to identify and define role and resource gaps to the hospital stakeholders. Often used in the *define* and *measure* steps of the DMAIC method, a project charter provided a tool to organize and track project data, including background information, a problem statement, current business condition, business case, goal statements, quantitative metrics, and a time line with completion dates.^24^

The *analysis* step of the DMAIC method compared the beginning state of the project with the prospective interventional outcomes by using statistical assessments, comparative outcome charts, and root cause analysis.^24^ The *improve* step was implemented in 2 phases, as depicted in Figure 1. Staff training and reallocation provided a solution to improve role and resource gaps. At the end of the process, improvement was implemented by adjusting to potential system breakdowns and future recommendations made by the leadership team to accommodate systemwide changes.

Finally, the *control* step prevented the initial problems from reverting back to the original conditions. Leadership buy-in for the final phase of the DMAIC method is critical for long-term project success.^25^ Establishing new preprocedure and recovery workflows, permanent staffing movements and on-going training were included among the CNS's responsibilities to ensure future preregistration, cardiac catheterization laboratory, and recovery area stability.

## METHODS

To provide adequate oversight and ethical integrity, the QI project was successfully submitted through a double internal review board process with the hospital and the author's academic institution. Data collection was conducted retrospectively. During the postintervention data collection period of 3 months, the CNS collected data from 107 eligible patients. Subsequently, 3 months of baseline data were collected between November 1, 2015, and February 1, 2016, from 71 patients with equivalent inclusion criteria. All electronic data were encrypted and password protected, and paper records were kept in locked cabinets in a secured location. To preserve patient confidentiality, protected health information was only used to determine the appropriate phases of care and whether the inclusion criteria were met, but no protected information was included in the final collated data.

### Participants

Participants were selected using a convenience sample. All adult outpatients with scheduled coronary angiograms were given an oral consent sheet, including project information, during their registration process. To determine the efficacy of the recovery nurse skill training program, only patients undergoing PCI and receiving manual compression to the procedural puncture site were included. Patients receiving intraoperative hemostatic closure devices were excluded. Over a 3-month period, the goal was to include at least 100 patients for an adequately sized data set.^26^

### Settings

Data collection took place in 3 settings. The first setting was within the preregistration unit, where nurses conducted prehospital telephone interviews. The interviews were completed in private offices to protect health information. All clinical information was entered into the hospital EHR.

The second setting was in Short Stay, which is the outpatient preprocedural area. Nurses working in the Short Stay unit prepared patients in semiprivate rooms after they were registered. Each patient was gowned and prepared for their procedure, which included peripheral intravenous line insertion, the provision of preprocedural medications, and screening for missing clinical information.

Finally, the third setting was the new post-PCI recovery unit that was adjacent to the cardiac catheterization laboratory. Close proximity to cardiologists and staff was intentionally orchestrated to increase comfort and communication among the recovery staff.^12^ Nine beds with advanced hemodynamic monitoring capabilities were made available in the new post-PCI recovery area to ensure that each nurse could adequately facilitate the recovery process in a safe and well-informed manner.

### Outcome Measures

There were 4 patient goal measurements used to evaluate the outcomes of the project. Three-month baseline values were calculated for each outcome and were used to compare project implementation improvements. A proposed mean reduction in preprocedural patient preparation times from 47.3 to 35 minutes addressed the first goal of streamlining the preregistration process. The second goal was to decrease costly PCI-related bleeding complications from 18% to 6% of patients per month through dedicated and well-trained nursing recovery staff. The third goal was to decrease the overall hospital length of stay from 1.35 to 1.0 days. Finally, the fourth goal was to elevate PCI patient satisfaction scores from 78% to 85% as evidenced by an enhanced continuum of care through staffing reallocation, process efficiency, and specialized PCI training, including the discharge process.^8^,^9^

### Intervention and Data Collection

The project interventions were driven by evidence that interdepartmental integration and training can close resource and data gaps.^3^,^15^ To address preregistration data gaps and the goals to reduce preprocedural preparation times and increase patient satisfaction, the CNS negotiated the provision of 2 dedicated preregistration nurses from Surgical Services, who were trained to conduct pre-PCI patient phone interviews. At least 1 full-time preregistration nurse was available each day to add PCI patients to their preregistration screening agenda. The preregistration nurses were already highly skilled to perform these tasks, and the acquisition of cardiac catheterization laboratory–specific knowledge required minimal education.

To measure the preregistration nurses' effectiveness, preprocedural preparation times in the Short Stay area were tabulated from the hospital EHR event tracking system. To prevent inappropriately long tracking times, nurses assigned to Short Stay were instructed to promptly update the patient EHR status to denote when they were ready for their procedure. The success of the intervention within the preregistration area was evidenced by a reduction in Short Stay patient-preparation times.

In reference to gaps in recovery resources and the goals to reduce bleeding complications, lower hospital length of stay, and increase patient satisfaction, the CNS acquired a previously underused but suitable space for the new post-PCI recovery area to accommodate additional PCI patient volumes. Not only was the new space adjacent to the cardiac catheterization laboratory, but it also provided an expedient and cost-effective implementation solution. For PCI recovery, patient monitoring equipment was already available within the new area for the nursing staff to use.

The CNS and a nursing cardiac catheterization educator jointly trained 8 additional postanesthesia recovery nurses to staff the post-PCI recovery area on rotating shifts during the day. Training included hemodynamic monitoring, manual and mechanical PCI puncture site hemostasis, proper patient positioning, and discharge teaching that was designed to increase patient self-awareness and reduce postprocedural complications. Percutaneous coronary intervention recovery training was conducted 8 hours per week over a period of 3 weeks, which allowed for flexibility within staff scheduling. The cardiac catheterization educator observed and reported PCI recovery practices as a part of their normal duties during the project data collection period. Despite the amount of education and skill building, mitigating at least 1 additional PCI complication had the potential to initially net a $5000 savings, which can increase stakeholder buy-in for an on-going training program.^6^,^10^,^11^

Qualifying bleeding complications, such as frank access site bleeding, serosanguinous oozing, hematomas, pseudoaneurisms, and vascular repairs during the same hospital visit were abstracted from the National Cardiovascular Database Registry.^27^ The Society for Cardiac Angiography and Interventions had also previously recommended this registry as a reliable repository for PCI data.^28^ The hospital had already been using the registry to track bleeding complications, which provided a stable source for data collection and reporting without having to change or create new processes for the project.

The combined efforts of reducing preprocedural preparation times and bleeding complications also contributed to the project goal of lowering the mean hospital length of stay for each patient. Mean length of stay was tracked and calculated through the EHR admission-discharge-transfer system. All movement activities, from registration to discharge, could be easily found on the patient's EHR summary screen.

Finally, the culmination of reducing preprocedural preparation times, mitigating bleeding complications, and lowering hospital length of stay merged to meet the goal to increase patient satisfaction scores. Composite outpatient satisfaction scores were collected from a national standardized postdischarge survey questionnaire that was conducted through the hospital's resources. On a scale of 1 to 10, scores of 9 and 10 for all questions were considered favorable responses. Favorable responses were tabulated into the numerator for each question to produce an overall patient satisfaction score.^9^

### Analysis

The registry data and admission-discharge-transfer reports were collated, cleaned, validated, and entered by the CNS into a SPSS (IBM Corp, Armonk, New York) database for analysis. The CNS performed a 2-tailed Pearson *r* coefficient analysis on 3 variables, which included preoperative patient preparation times, hospital length of stay, and the occurrence of bleeding complications. The comparison was designed to identify any relationships among the variables with a significance level of .01 or better.

## RESULTS

**FIGURE 2.**
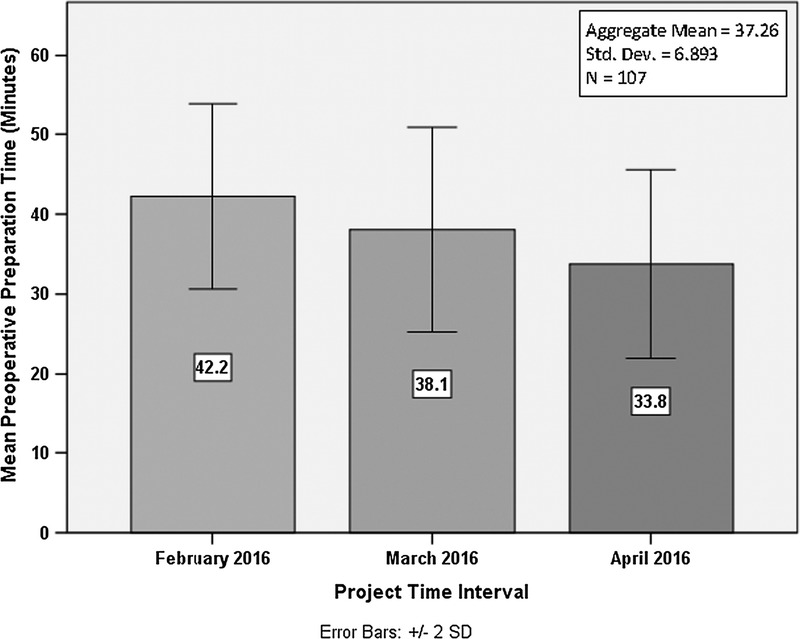
Progression of mean preoperative preparation times (postproject intervention).

There were noticeable improvements observed in all 4 project goals, which were to reduce preprocedural preparation times, reduce the rate of bleeding complications, decrease outpatient PCI patient hospital length of stay, and increase overall patient satisfaction. The monthly trends for preoperative preparation times and hospital length of stay were highly encouraging. The goal to reduce preoperative patient preparation times to 35 minutes was met in the final month of the project implementation period by dropping the baseline average of 47.3 minutes to 33.8 minutes, with an aggregate mean of 37.26 minutes for the entire 3-month period. Figure 2 illustrates a steady downward trend with a consistent variance in observed times.

After examining the 3-month project implementation data, only a mean of 6.54% patients experienced a bleeding complication per month. A nearly 3-fold reduction in bleeding was observed when compared with more than 18% per month from the 3-month baseline period. When comparing the baseline and project implementation periods, 12 patients were potentially spared from procedural insertion site complications.^27^,^29^

**FIGURE 3.**
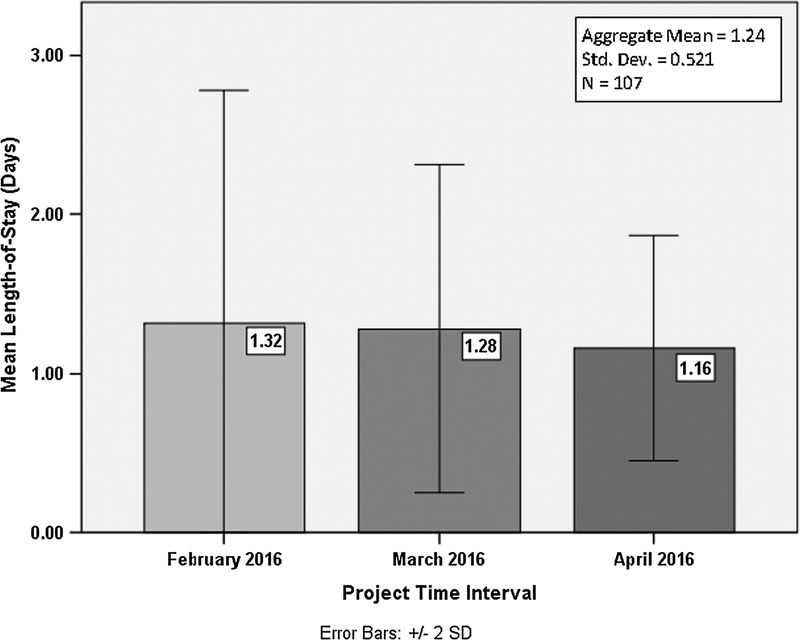
Progression of mean hospital length of stay (post-project intervention).

Hospital length of stay dropped from a 3-month baseline mean of 1.35 days to 1.24 days with a similar downward trend seen with preprocedural preparation times. By the final month of the project intervention period, the mean number of hospital days settled at 1.16 days. Although the goal to reduce total outpatient PCI hospital time to 1 day was not entirely met, the variance in total time stayed was reduced each successive month during the project implementation period, as illustrated in Figure 3.^29^

Finally, there was a noticeable improvement in patient satisfaction scores when compared with the baseline data. The final composite score of favorable responses rose from 78% to 86.5%, which exceeded the project goal of 85%. The responses were specifically tabulated from the Short Stay phase of care and from a general pool of questions representing the outpatient perioperative PCI areas.^29^

**FIGURE 4.**
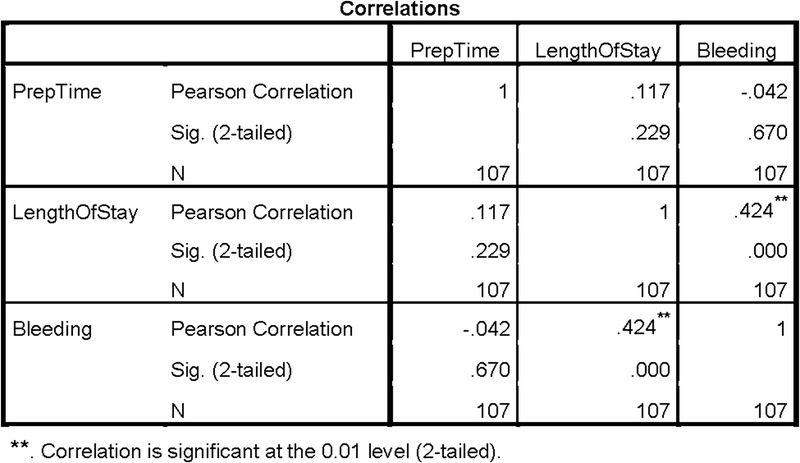
Correlation between preoperative preparation time, hospital length of stay, and bleeding occurrences.

After comparing patient preparation times, length of stay, and bleeding occurrences with a Pearson *r* correlation analysis, only 1 set of variables displayed a relationship as shown in Figure 4. A moderate positive correlation was revealed (*r* [105] = 0.424, *P* < .001), indicating that increased hospital lengths of stay were related to post-PCI bleeding events among the sampled patient participants.^30^ Although the correlation was statistically moderate, it validated a noticeable connection to PCI recovery practices.

**Table:**
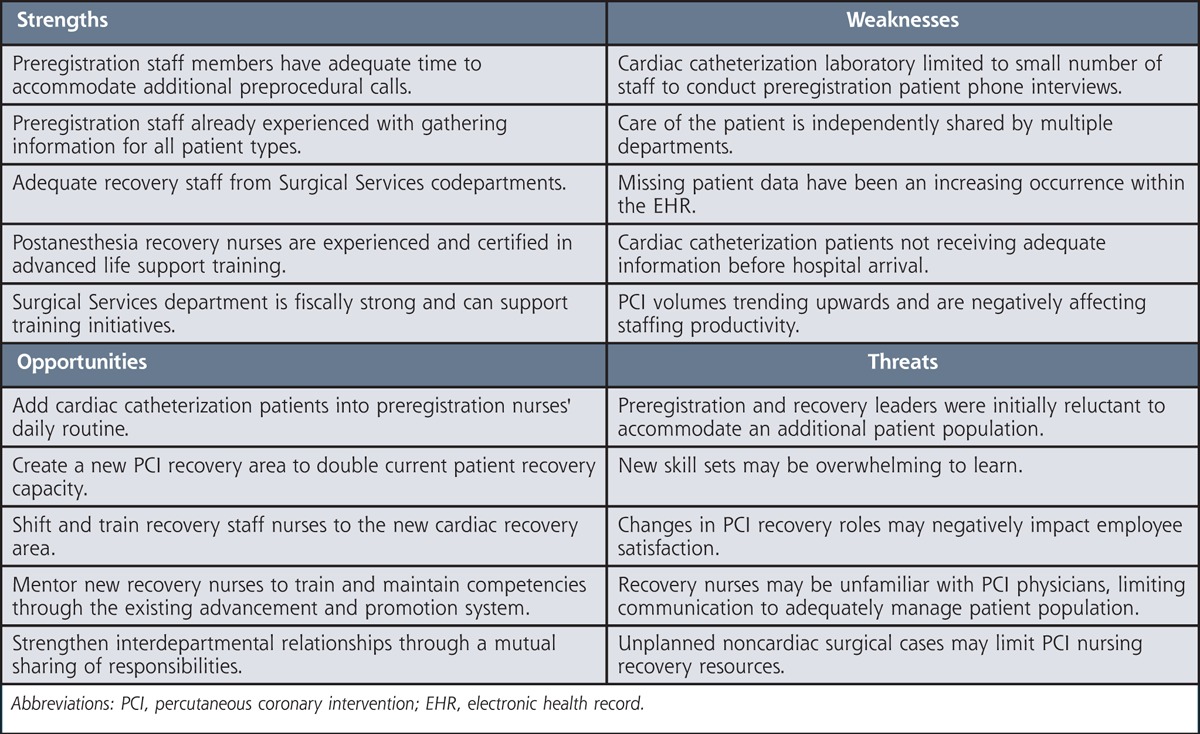
Perioperative SWOT Analysis

## DISCUSSION

In review, the purpose of this project was to provide an efficient, safe, and satisfying PCI patient experience with flexible and knowledgeable staff, who can adapt to growing procedural volumes. The most dramatic accomplishment was a reduction in preoperative patient preparation times. A large amount of the improvement derived from equally valued formative or process-related changes in 2 distinct phases of care.^31^ Addressing the first phase of care within the preregistration unit was essential to relieving the cardiac catheterization staff from gathering preoperative patient information and providing prehospital instructions. Although the preregistration nurses were already well trained to conduct prehospital patient interviews, the CNS and unit leadership were instrumental in ensuring that preprocedural PCI patient orders, procedural contraindications, and laboratory instructions were well established and communicated. The preregistration nurses and the CNS also collaborated by helping leadership to understand the differences between standard surgical and PCI preparation. Historically, one of the largest mistakes in reassigning preoperative clinical staff functions was in making assumptions that all procedural patients have the same needs and requirements.^3^,^15^,^20^ A preoperative PCI reference guide was subsequently created for the preregistration nurses to follow as patient needs and medical procedures changed. Research has demonstrated that revisiting processes with practice checklists or refined policies can be beneficial to nursing recall and role-based confidence.^5^,^6^,^15^,^20^

Although the QI project did not compare the quality and quantity of patient data recovered by the preprocedural role reallocation process, the nurses in the preoperative Short Stay area provided valuable qualitative feedback regarding the completeness of patient data in the EHR. Instead of gathering original data for the hospital EHR, the nurses in Short Stay reported that they saved time by simply verifying preobtained data. From a safety perspective, the chance for harmful errors is reduced during in-hospital preprocedure preparation when potentially nervous, distracted, or medically compromised patients can avoid multiple and redundant clinical inquiries for personal information.^3^,^32^ In conjunction, the CNS and preregistration nurses improved the preoperative PCI process by asking patients to bring a copy of their history and medications to enhance the verification process. In total, the overall 21.2% drop in patient preparation times was attained with the innovative collaboration and cooperation among the preoperative staff.

Despite a nearly 50% increase in PCI volumes from the baseline period to the end of the QI project intervention, the number of bleeding complications were reduced to nearly one-third of the baseline figures.^8^ The significant decrease resulted from a combination of staffing and hospital infrastructure changes. Not only the reallocation of existing postanesthesia recovery nurses proved to be a valuable resource to reduce training and new-hire orientation costs, but also the experienced staff only required a small amount of specialized PCI recovery training. The presence of hemodynamic monitoring equipment was not a hindrance, because the nurses were accustomed to monitoring in the postanesthesia units. The largest learning curve was addressed during procedural access site hemostasis training and the delivery of discharge teaching knowledge to patients in the recovery unit. Although the nurses performed approved recovery practices, they were encouraged to voice clinical preferences, such as choosing the most comfortable manual compression methods, especially when patients required prolonged arterial pressure to maintain access site hemostasis. Staffing input also boosts employee satisfaction and compliance and lead to a reduction in bleeding complications.^4^,^13^,^15^,^19^ In parallel, discharge-teaching practices were also enhanced through staff-inspired reference sheets, which doubled as informational pamphlets for the patients. Rich with illustrations and simple explanations, the pamphlets allowed teaching with patients of varying learning needs and abilities.

In conjunction with the recovery nurse training, the location of the newly acquired PCI recovery area near the cardiac catheterization laboratory provided additional enhancements to cross-communication and recovery nurse confidence. However, during the project implementation period, the PCI recovery area was only used for 50% of the time, largely because of area-adjacent construction activities. Despite the complication in area logistics, the recovery nurses proved to be flexible, adaptable, and able to rely on their training and leadership.

Regarding length-of-stay results, there was a sharp increase in PCI volumes in the last month of the project implementation phase. Each cardiologist also conducted varying practices for post-PCI discharge criteria, such as recovery holding times, but these windows rarely varied more than 6 hours, unless a bleeding complication arose.^32^ If a particular physician allows for same-day PCI discharges in higher-than-normal volumes, aggregate results can become skewed.

Although one's hospital length of stay can be influenced by multiple factors, evidence suggests that PCI-related bleeding complications contribute to a large part of this time frame to allow for additional monitoring or to mitigate further complications.^4^,^13^,^17^,^19^ Each patient's hospital admission was determined from the time that they arrived and registered for their PCI procedure until they were discharged either home or to a designated care facility. Statistical analysis of the data from the project also confirmed that post-PCI bleeding represented the most significant finding. Three patients presented with frank bleeding, and 4 patients experienced serosanguinous oozing from 2 to 12 hours after their PCI procedures. However, femoral sheath sizes and specific nursing hemostasis techniques were not used as variables for data analysis. The average length of stay for the 7 individuals who experienced a bleeding complication during the project intervention period was 2.32 days, which suggested a link to bleeding and increased hospital stays. Interestingly, there was no discernable pattern of improvement for bleeding complications over time to reflect patient teaching, which can be attributed to a relatively short intervention and data collection period.^17^,^21^

Evidence suggests that patients discharged from the hospital at shorter intervals with less discomfort or complications show greater satisfaction with care.^4^,^16^,^33^ The final patient satisfaction score of 86.5% was reflective of the newly streamlined and accommodating PCI process. In observance of patient satisfaction scores, the responses may have reflected a general perspective, rather than being based on individual experiences.^26^ Only aggregate scores could be obtained, instead of granular data, which may have revealed a greater diversity in patient opinions.

In addition to time- and comfort-related improvements, patient education also enhances a sense of self-efficacy and trust with clinical providers.^34^ As a result, patients become more receptive to complying with prescribed regimens and thus exhibit improved long-term outcomes.^21^,^35^ The CNS also elicited additional staffing buy-in through one-on-one postintervention evaluation and feedback sessions to enhance the perioperative nurses' sense of ownership to the process. As evidenced by this project and supported by research, well-invested clinicians are more willing to deliver a better patient experience, which will generate a higher level of patient satisfaction.^16^,^20^,^21^

## CONCLUSION

With a project purpose to streamline and improve a growing PCI program, the 3-month postinterventional trends showed successive improvements to patient outcomes, hospital throughput, and communication between preprocedural staff, patients, physicians, and recovery staff. The originally proposed goals for preprocedural preparation times, bleeding complications, and patient satisfaction were met by the end of project implementation period. Although the predefined goal for hospital length of stay was not numerically met, the overall average time was reduced, including the variance in total days stayed, which provided greater consistency to admission durations.

The QI-based DMAIC method provided structure to identify, track, measure, and regulate the project goals while also serving as a familiar tool for clinical and business-oriented stakeholders. The resulting process flowchart provided a visual aid to help the CNS and stakeholders stay on track and avoid unnecessary duplication or diversions. Overall, the use of a QI model increased project buy-in and stimulated a higher level of leadership participation.

Reallocating staff roles among related interdepartmental service lines, including preregistration phone interviews and post-PCI recovery services provided a cost-effective, efficient, and safer patient experience. Although the CNS provided specific training to fill knowledge gaps, the newly assigned roles were already a part of the nurses' basic skill sets. Introducing nurses who have no previous perioperative knowledge will likely cause project delays and initially introduce unfavorable results.

Ultimately, the CNS served as a leader and project champion to facilitate significant changes in a system of care that had many resource and data gaps. Collectively uniting the perioperative PCI continuum of care proved to be a feasible endeavor and had a positive effect on patient satisfaction and outcomes. Interdepartmental collaboration strengthened clinical resources and provided a more cohesive system of care that focused on long-term clinical success.

## References

[1] Grüntzig A Transluminal dilatation of coronary-artery stenosis. *Lancet*. 1978;1(8058):263. 74678 10.1016/s0140-6736(78)90500-7

[2] Crouse SB Kitko LA Outcomes of coronary artery interventions: comparing coronary artery bypass surgery and percutaneous coronary intervention in patients with unprotected left main stenosis. *J Am Assoc Nurse Pract*. 2014;26(2):91–101. 24170643 10.1002/2327-6924.12078

[3] Kesavan S Kelay T Collins RE Clinical information transfer and data capture in the acute myocardial infarction pathway: an observational study. *J Eval Clin Pract*. 2013;19(5):805–811. 22587539 10.1111/j.1365-2753.2012.01853.x

[4] Chhatriwalla AK Amin AP Kennedy KF Association between bleeding events and in-hospital mortality after percutaneous coronary intervention. *JAMA*. 2013;309(10):1022–1029. 23483177 10.1001/jama.2013.1556

[5] Hamric AB Hanson CM Tracy MF O’Grady ET *Advanced Practice Nursing: An Integrative Approach*. 5th ed St Louis, MO: Saunders Elsevier; 2014.

[6] Fulton JS Lyon BL Goudreau KA *Foundations of Clinical Nurse Specialist Practice*. 2nd ed New York, NY: Springer Publishing Company; 2014.

[7] California Legislative Information. SB-906 elective percutaneous coronary intervention (PCI) program. California Legislative Information Web site. http://leginfo.legislature.ca.gov/faces/billNavClient.xhtml?bill_id=201520160SB906. Accessed March 11, 2016.

[8] National Cardiovascular Data Registry. Registries. https://cvquality.acc.org/NCDR-Home/registries. Accessed June 1, 2016.

[9] Hospital Consumer Assessment of Healthcare Providers and Systems. Survey Instruments. 2015 http://www.hcahpsonline.org/surveyinstrument.aspx. Accessed March 11, 2016.

[10] Kaiser Family Foundation. Hospital adjusted expenses per inpatient day. Kaiser Family Foundation Web site. http://kff.org/other/state-indicator/expenses-per-inpatient-day/. Accessed March 12, 2016.

[11] State of California Office of Statewide Health Planning & Development. Clovis community medical center charge master as of 06/01/15. 2015. https://www.oshpd.ca.gov/Chargemaster/Hospitals/2015/Clovis%20Community%20Medical%20Center/106100005_CDM_All_2015.xls.xls. Accessed March 12, 2016.

[12] Hall HR Roussel LA *Evidence-Based Practice: An Integrative Approach to Research, Administration, and Practice*. Burlington, MA: Jones and Bartlett; 2014.

[13] Dauerman HL Rao SV Resnic FS Applegate RJ Bleeding avoidance strategies. Consensus and controversy. *J Am Coll Cardiol*. 2011;58(1):1–10. 21700085 10.1016/j.jacc.2011.02.039 PMC3127231

[14] Centre for Evidence-based Medicine. Oxford centre for evidence-based medicine—levels of evidence. Centre for Evidence-based Medicine Web site. http://www.cebm.net/oxford-centre-evidence-based-medicine-levels-evidence-march-2009/. Accessed January 16, 2016.

[15] Rolley JX Salamonson Y Dennison CR Davidson PM Development of clinical practice guidelines for the nursing care of people undergoing percutaneous coronary interventions: an Australian & New Zealand collaboration. *Austr Crit Care*. 2010;23(4):177–187. 10.1016/j.aucc.2010.03.004 20413321

[16] Rolley JX Davidson PM Salamonson Y Fernandez R Dennison CR Review of nursing care for patients undergoing percutaneous coronary intervention: a patient journey approach. *J Clin Nurs*. 2009;18(17):2394–2405. 19538559 10.1111/j.1365-2702.2008.02768.x

[17] Higgins M Theobald K Peters J Vascular access and cardiac complications after PCI: in- and out-of-hospital outcome issues. *Br J Card Nurs*. 2008;3(3):111–116.

[18] Mert H Seren Intepeler S Bengu N Baturlar Z Istan P Ozcelik E Efficacy of frequent blood pressure and heart rate monitoring for early identification of bleeding following percutaneous coronary intervention. *Int J Nurs Pract*. 2012;18(1):52–59. 22257331 10.1111/j.1440-172X.2011.01984.x

[19] Tavris DR Wang Y Jacobs S Bleeding and vascular complications at the femoral access site following percutaneous coronary intervention (PCI): an evaluation of hemostasis strategies. *J Invasive Cardiol*. 2012;24(7):328–334. 22781471

[20] Kilonzo B O'Connell R Secondary prevention and learning needs post percutaneous coronary intervention (PCI): perspectives of both patients and nurses. *J Clin Nurs*. 2011;20(7):1160–1167. 21385251 10.1111/j.1365-2702.2010.03601.x

[21] Hu X Zhu X Liu Y Gao L Intensive nursing care by an electronic followup system to promote secondary prevention after percutaneous coronary intervention. *J Cardiopulm Rehabil Prev*. 2014;34(6):396–405. 24667664 10.1097/HCR.0000000000000056

[22] Berry T What is a SWOT analysis?. BPlans Web site. http://articles.bplans.com/how-to-perform-swot-analysis/. Accessed March 13, 2016.

[23] Harris JL Roussel L Walters SE Dearman C *Project Planning and Management: A Guide for CNLs, DNPs, and Nurse Executives*. Sudbury, MA: Jones & Bartlett; 2011.

[24] Moran K Burson R Conrad D *The Doctor of Nursing Practice Scholarly Project: A Framework for Success*. Burlington, MA: Jones & Bartlett; 2014.

[25] iSixSigma. Six sigma DMAIC roadmap. iSixSigma Web site. http://www.isixsigma.com/new-to-six-sigma/dmaic/six-sigma-dmaic-roadmap/. Accessed January 15, 2016.

[26] Polit DF Beck CT *Nursing Research: Generating and Assessing Evidence for Nursing Practice*. 9th ed Philadelphia, PA: Lippincott Williams & Wilkins; 2012.

[27] National Cardiovascular Data Registry. Coder’s data dictionary V4.4. National Cardiovascular Data Registry Web site. https://www.ncdr.com/WebNCDR/docs/public-data-collection-documents/cathpci_v4_codersdictionary_4-4.pdf. Accessed March 11, 2016.

[28] Dehmer GJ Blankenship JC Cilingiroglu M SCAI/ACC/AHA expert consensus document: 2014 update on percutaneous coronary intervention without on-site surgical backup. *Circulation*. 2014;129(1):2610–2626. 24637561 10.1161/CIR.0000000000000037

[29] National Research Corporation. CCMC OPS patient satisfaction trend graphs: 2016 https://nrcpicker.com/eReports/SignIn.aspx?ReturnUrl=%2feReports%2fDefault.aspx. Accessed June 12, 2016.

[30] Cronk B *How to Use SPSS®: A Step-by-Step Guide to Analysis and Interpretation*. 8th ed Glendale, CA: Pyrczak Publishing; 2014.

[31] Pender NJ Murdaugh CL Parsons MA *Health Promotion in Nursing Practice*. 7th ed Upper Saddle River, NJ: Pearson; 2015.

[32] Haynes AB Weiser TG Berry WR A surgical safety checklist to reduce morbidity and mortality in a global population. *N Engl J Med*. 2009;360:491–499. 19144931 10.1056/NEJMsa0810119

[33] Falcone AM Bose R Stoler RC The ambulatory closure device percutaneous intervention (ABCD-PCI) study: a single-center experience. *Proc (Bayl Univ Med Cent)*. 2011;24(3):192–194. 21738289 10.1080/08998280.2011.11928713 PMC3124901

[34] de Chesnay M Anderson BA *Caring for the Vulnerable: Perspectives in Nursing Theory, Practice, and Research*. 4th ed Burlington, MA: Jones & Bartlett; 2016.

[35] Butts JB Rich KL *Philosophies and Theories for Advanced Nursing Practice*. 2nd ed Burlington, MA: Jones & Bartlett; 2015.

